# Human Disseminated Protothecosis: The Skin is the “Window”?

**DOI:** 10.3389/fimmu.2022.880196

**Published:** 2022-06-14

**Authors:** Xue Wang, Yuanshuai Ran, Songgan Jia, Sarah Ahmed, Xuemei Long, Yinhui Jiang, Yanping Jiang

**Affiliations:** ^1^ Department of Dermatology, The Affiliated Hospital of Guizhou Medical University, Guiyang, China; ^2^ Department of Microbiology, Basic Medical School, Guizhou Medical University, Guiyang, China; ^3^ Centre of Expertise in Mycology of Radboud University Medical Center/Canisius Wilhelmina Hospital, Nijmegen, Netherlands; ^4^ Laboratory of Medical Molecular Biology, Guizhou Medical University, Guiyang, China

**Keywords:** humans, immunosuppression, skin diseases, diagnostic errors, *Prototheca*

## Abstract

Human disseminated protothecosis is a rare infection caused by members of the genus *Prototheca*, an achlorophyllic algae always associated with debilitated hosts. The presence of non-budding cells and large, spherical cells (sporangia) with endosporulation (morula) in histology is proof of *Prototheca* infection. Regrettably, due to the lack of specificity of clinical features and low awareness among clinicians, protothecosis is always underestimated and misdiagnosed. The available data on a species-specific analysis of this infection are limited. In this review, we summarize the etiological, epidemiological, and clinical aspects of disseminated protothecosis. The potential pathogenicity and clinical differences between *P. zopfii* and *P. wickerhamii* were observed. Additionally, the skin not only became the main invasion site but also the most involved organ by the pathogen. With the increasing numbers of immunocompromised individuals throughout the world, the incidence of disseminated infection caused by *Prototheca* is bound to increase, and disseminated protothecosis that accompanies skin symptoms should be taken into account by clinicians.

## Introduction

In humans, disorders caused by *Prototheca* species can be cutaneous, olecranon bursitis, systemic, or disseminated (1). In contrast to the former two types of infection, disseminated protothecosis is mainly associated with immunocompromised hosts, such as patients under immunosuppressive therapy or with longstanding intravascular catheters, cancer, AIDS, diabetes mellitus or solid organ transplantation (2). This infection type had the worst prognosis, with only 33% cure or improvement, and 56% death (1). Thus, characterized by high mortality rates and with the increasing numbers of immunocompromised individuals throughout the world, disseminated protothecosis has aroused a gradual interest in the study of the various aspects of this infection and its causative microorganism.

Regrettably, due to the lack of specificity of clinical features and the lack of awareness among clinicians, up to date, only around 200 cases of *Prototheca* infection have been reported worldwide and systemic infection cases account for about 9% of the cases (1). Moreover, even after the start of the phycology working group in ISHAM (The International Society for Human & Animal Mycology) in 2017, little is known about *Prototheca* spp. or the disease caused by these algae, especially the disseminated type (1). Also, whether there are possible differences in pathogenicity within or between *Prototheca* species compared to other types of infection? This review provides a summary of the literature addressing etiological, epidemiological, and clinical aspects of disseminated protothecosis. According to a state defined by the presence of an achlorophyllic alga pathogen in the blood (algaemia) and/or any other sterile deep-tissues, organs, or there are more than 2 lesions in the whole body (1, 3), to our knowledge, 37 cases have been described in the literature from 1970 to 2019 with disseminated protothecosis.

## Methods

We searched the PubMed database by using the terms “disseminated protothecosis”, “systemic protothecosis”, “human”, and “Prototheca”. We only reviewed cases with sufficient clinical information and laboratory/sequence data to identify the alga in the period 1970–2019. Of these, 35 articles presenting 37 cases of human disseminated/systemic protothecosis were included (**Table 1**) (3–37).

**Table 1:** Clinical description of patients with disseminated protothecosis collected during the study period.

Patient	Year	Age	Sex	Country	Underlying condition	Immunity state	Neutropenia	Infectious site	Initial symptom	Species	Coinfection	Sample	Treatment	Outcome	Ref
1	1974	29	Male	New Zealand	Healthy	Immunocompetent	No	Skin, blood, liver	Skin papules, jaundice, diarrhea	*P. wickerhamii*	N/A	Skin	AMB	Cure	(3)
2	1978	30	Male	USA	Liver transplant	Immunocompromised	N/A	Skin	Skin papules	*P. wickerhamii*	*Candida albicans*, *Proleus mirabilis*, Klebsiella	Skin	None	Death	(4)
3	1986	41	Female	Australia	CAPD	Immunocompromised	N/A	Peritoneum	Abdominal pain	*P. wickerhamii*	N/A	Dialysate	AMB	Cure	(5)
4	1990	39	Male	USA	Healthy	Immunocompetent	No	Liver, gut	Abdominal pain, nausea	*P. wickerhamii*	N/A	Stool	AMB	Cure	(6)
5	1990	72	Male	USA	CAPD	Immunocompromised	N/A	Peritoneum	Abdominal pain, Fever	*P. wickerhamii*	N/A	Dialysate	AMB	Cure	(7)
6	1991	7	Male	USA	Leukemia	Immunocompromised	N/A	Blood	Fever	*P. wickerhamii*	*Pseudomonas aeruginosa*	Blood	AMB	Cure	(8)
7	1991	45	Male	England	CAPD	Immunocompromised	N/A	Peritoneum	Abdominal pain	*P. wickerhamii*	N/A	Dialysate	FLC	Cure	(9)
8	1992	24	Female	USA	Diabetes mellitus	Immunocompetent	No	Nasopharynx, esophagus	Nausea, vomiting	*P. wickerhamii*	*Staphylococcus aureus*	Esophageal and Nasopharyngeal lesion	AMB	Cure	(10)
9	1992	13	Male	Japan	Anemia	Immunocompetent	N/A	Gut, liver	Fever	*P. wickerhamii*	N/A	Stool	AMB	Cure	(11)
10	1992	80	Male	Japan	Diabetes mellitus	Immunocompetent	N/A	Skin	Skin papules	*P. wickerhamii*	N/A	Skin	KET	Cure	(11)
11	1992	25	Female	USA	AIDS, Skin trauma	Immunocompromised	No	Brain	Fever, headache	*P. wickerhamii*	*Cryptococcus neoformans*	CSF	AMB	Death	(12)
12	1996	59	Female	USA	Lung transplant	Immunocompromised	N/A	Blood	Fever	*P. zopfii*	N/A	Blood	FLC	Death	(13)
13	1996	20	Male	Japan	Anemia	Immunocompetent	N/A	Brain	Fever, headache	*P. wickerhamii*	N/A	CSF	AMB	Cure	(14)
14	1997	75	Male	USA	Myasthenia gravis	Immunocompromised	N/A	Blood, skin	Skin papules, fever	*P. wickerhamii*	N/A	Blood	FLC	Cure	(15)
15	1998	36	Male	Israel	Candidiasis	Immunocompromised	N/A	Gut	Abdominal pain	*Prototheca* spp.	*Candida albicans*	Colon tissue	ITC	Cure	(16)
16	2002	39	Male	USA	Adenocarcinoma	Immunocompromised	N/A	Lung	Fever	*Prototheca* spp.	N/A	Bronchoalveolar lavage	FLC	Death	(17)
17	2002	19	Male	USA	Stem cell transplant	Immunocompromised	N/A	Blood	Fever	*Prototheca* spp.	N/A	Blood	AMB	Cure	(17)
18	2004	56	Male	USA	Stem cell transplant	Immunocompromised	N/A	Blood, skin	Skin papules, fever	*P. wickerhamii*	*Klebsiella pneumoniae*	Blood	AMB	Death	(18)
19	2004	49	Male	USA	AIDS	Immunocompromised	N/A	Blood, skin	Fever, swelling of skin	*P. wickerhamii*	N/A	Skin	AMB	Death	(19)
20	2004	58	Male	Australia	Stem cell transplant	Immunocompromised	N/A	Blood, skin	Skin papules, fever	*P. zopfii*	N/A	Blood	VRC, AMB	Death	(20)
21	2005	58	Male	USA	Stem cell transplant	Immunocompromised	No	Blood, skin, lung, liver	Skin papules	*P. wickerhamii*	N/A	Blood	AMB	Death	(21)
22	2007	24	Male	China	Healthy	Immunocompetent	No	Brain	Headache	*P. wickerhamii*	N/A	CSF	AMB, ITC	Cure	(22)
23	2008	61	Male	USA	Liver transplant	Immunocompromised	No	Blood, skin	Skin papules	*P. wickerhamii*	*Escherichia coli*	Blood	AMB	Death	(23)
24	2008	10	Male	India	Skin trauma	Immunocompromised	Yes	Skin, spleen	Skin papules	*P. wickerhamii*	N/A	Skin	AMB	Cure	(24)
25	2010	49	Female	England	Leukemia	Immunocompromised	Yes	Blood, skin	Fever, skin necrosis	*P. wickerhamii*	*Enterococcus faecium*	Skin, blood	VRC	Cure	(25)
26	2011	78	Female	Australia	Cardiac transplant	Immunocompromised	N/A	Blood, skin	Skin papules, fever	*P. wickerhamii*	N/A	Blood	AMB, ITC	Death	(26)
27	2012	61	Male	Malaysia	Renal transplant	Immunocompromised	N/A	Blood	Fever	*P. wickerhamii*	N/A	Blood	None	Death	(27)
28	2013	2	Female	Mexico	Healthy	Immunocompetent	N/A	Skin	Skin abscess, fever	*Prototheca* spp.	*Blastomyces dermatitidis*	Skin	ITC	Cure	(28)
29	2014	4	Female	Singapore	Liver transplant	Immunocompromised	No	Blood, skin	Skin papules, fever	*P. wickerhamii*	N/A	Blood	AMB	Cure	(29)
30	2014	74	Male	USA	Leukemia	Immunocompromised	N/A	Skin	Skin papules	*P. wickerhamii*	N/A	Skin	ITC	Cure	(30)
31	2014	56	Female	Australia	Stem cell transplant	Immunocompromised	N/A	Blood, skin	Fever, skin cellulitis	*P. zopfii*	N/A	Blood	AMB	Death	(31)
32	2015	46	Female	Japan	Leukemia	Immunocompromised	Yes	Skin	Skin papules	*P. zopfii*	N/A	Skin	None	Death	(32)
33	2018	36	Male	India	Liver transplant	Immunocompromised	N/A	Blood, skin, lung	Skin papules, chest pain	*P. zopfii*	Klebsiella	Blood	AMB	Death	(33)
34	2018	8	Female	Turkey	Inherited CARD9 deficiency	Immunocompromised	No	Gut	Abdominal pain, diarrhea	*P. zopfii*	N/A	Blood	AMB	Cure	(34)
35	2018	19	Male	Spain	Leukemia	Immunocompromised	Yes	Blood	Fever	*P. zopfii*	N/A	Blood	AMB	Death	(35)
36	2019	31	Male	Morocco	Candidiasis	Immunocompromised	N/A	Gut	Abdominal pain diarrhea	*P. wickerhamii*	*Candida albicans*	Colon tissue	FLC	Death	(36)
37	2019	13	Male	China	Leukemia	Immunocompromised	Yes	Blood	Skin papules fever	*P. zopfii*	N/A	Blood	FLC	Death	(37)

CAPD, chronic ambulatory peritoneal dialysis; AIDS, Acquired Immune Deficiency Syndrome; CSF, cerebrospinal fluid;AMB, amphotericin; FLC, fluconazole; VRC, voriconazole; ITC, itraconazole; KET, ketoconazole.

N/A, Data is not available.

Charts were reviewed for clinical details. Data collected included demographics, location, risk factors, affected site, causative species, diagnostic investigations, management, and outcomes. The χ^2^ test was used for comparisons of categorical variables with a level of significance of.05, and the Fisher exact test was used in the analysis of contingency tables when the sample sizes were small. All analyses were conducted with SPSS version 22.0 (IBM Corp., Armonk, New York).

## Etiology

Originally, *Prototheca* was described in 1894 as a yeast-like fungus recovered from slime flux in trees (38). However, in 1913, and on the basis of ultra-structure, life cycle, and reproduction system, the genus was recognized as algae (39). It was thought to be a mutant of the genus *Chlorella* that is unable to produce chlorophyll and lacks photosynthesis. Currently, *Prototheca* is accepted as a separate genus in the family *Chlorellaceae* and comprises unicellular algae that reproduce asexually *via* sporangia with sporangiospores. Eight species are known, i.e., *P. blaschkeae*, *P. cutis*, *P. miyajii*, *P. wickerhamii*, *P. zopfii*, *P. stagnora*, *P. ulmea*, and the recently described *P. tumulicola* (40). The former five have been associated with human and animal infection *P. wickerhamii* was the predominate one. Only *P. zopfii* and *P. wickerhamii* have been reported to cause disseminated infection in humans.

Out of the 37 cases of disseminated protothecosis reported in the literature, 25 were caused by *P. wickerhamii* (67.6%), 8 by *P. zopfii* (21.6%), and for the remaining four cases (10.8%), the cause was not identified to the species level. Unlike *P. wickerhamii*, which can cause skin, blood, cerebrospinal, and gastrointestinal tract infections, *P. zopfii* appears to be primarily detected in the blood (6/8), with higher mortality (7/8, 87.5%) than *P. wickerhamii* (9/25, 36.0%) (*P* <.05) (**Table 2**). Whether this is related to the potential virulence or pathogenicity of different species is not yet known. A previous study has indeed demonstrated that *P. zopfii* is lethal for immunosuppressed mice compared to *P. wickerhamii*, which could not induce infection (2, 41). Thus, the scant number of *P. zopfii* infections might in fact be due to the lower abundance of this species in the environment rather than the low pathogenic potential of the species. Nevertheless, ecological studies on the distribution and abundance of *Prototheca* species have not been conducted so far, and other factors that might contribute to the lower frequency of *P. zopfii* infections could not be excluded.

**Table 2:** Presenting characteristics of patients with disseminated protothecosis. Values are numbers.

Characteristics	Total (37)	Death (17)	*P*-Value
Median Age, years	39.0 (2.0–80.0)	56.0 (13.0–78.0)	
<30years	14 (37.8%)	3 (21.4%)	
≥30years	23 (62.2%)	14 (60.9%)	<0.05
Sex			
Female	11 (29.7%)	5 (45.5%)	
Male	26 (71.1%)	12 (46.1%)	.969
Underlying condition			
Transplantation	12 (32.4%)	10 (83.3%)	<0.05
Renal transplant	1 (2.7%)	1 (100.0%)	
Lung transplant	1 (2.7%)	1 (100.0%)	
Liver transplant	4 (10.8%)	3 (75.0%)	
Cardiac transplant	1 (2.7%)	1 (100.0%)	
Stem cell transplant	5 (13.5%)	4 (80.0%)	
Candidiasis	2 (5.4%)	1 (50.0%)	
Myasthenia gravis	1 (2.7%)	0 (0%)	
Leukemia	6 (16.2%)	3 (50.0%)	
Anemia	2 (5.4%)	0 (0%)	
AIDS	2 (5.4%)	2 (100.0%)	
Diabetes mellitus	2 (5.4%)	0 (0%)	
Chronic ambulatory peritoneal dialysis	3 (8.1%)	0 (0%)	
Adenocarcinoma	1 (2.7%)	1 (100.0%)	
Skin trauma	1 (2.7%)	0 (0%)	
Inherited CARD9 deficiency	1 (2.7%)	0 (0%)	
Skin trauma/surgery/catheter-related^a^	22 (59.5%)	13 (59.1%)	
**Sign and symptoms at disease onset**			
Fever	20	11	
Skin papules	19	11	
Abdominal pain	7	1	
Headache	3	1	
Diarrhea	3	1	
**Species**			
* P. wickerhamii*	25 (67.6%)	9 (36.0%)	
* P. zopfii*	8 (21.6%)	7 (87.5%)	<0.05
* Prototheca* spp. (unidentified)	4 (10.8%)	1 (25.0%)	

Data are number/total number (%) unless indicated otherwise.

a Defined as the above causes of skin barrier destruction.

## Epidemiology

*Prototheca* species are saprophytes, occupying niches with decaying organic matter in moist environments, such as tree slime flux, animal manure, and sewage. They can also be found on plant and food item surfaces, in soil, or in water. In mammals, species can be present as transient gastrointestinal flora or as asymptomatic colonizers of skin and nails (2, 39, 42). From being considered non-pathogenic to causing disseminated infections in humans, we are rediscovering the mysterious algae ubiquitous in nature.

Prototheca infection could spread through exogenous or endogenous routes. The former is related to defects in the skin and mucosa (such as postoperative wounds). Compared to traumatic inoculation with the algae from the environment, dermal barrier destruction caused by hospital-acquired cases, including surgical operations and catheter-related procedures, are main exogenous route of invasion. *Prototheca* spp. may survive chlorination by forming biofilms (43) and be returned to the environment *via* sewage effluent and household waste. Cows with mastitis caused by *P. zopfii* (genotypes 1) may be a source for infection in humans, with immunocompromised farmers at the highest risk (44). Although the possibility of human-to-human transmission was raised with the outbreak of *P. wickerhamii* algaemia and sepsis in a tertiary care chemotherapy oncology unit recently (45), although contrary to our traditional understanding, the report cannot be ignored because of the public safety concerns. Endogenous colonization during prolonged immunosuppression in the gut followed by translocation resulting in algaemia and sepsis is suspected to be the cause of the outbreak.

Disseminated protothecosis is common in patients with underlying immunosuppression or several underlying diseases (2). Our review indicated that there were still four patients (10.8%) with normal immune status, but all of them were successfully treated and had a good prognosis, and most of the cases were associated with *P. wickerhamii* (3/4, 75.0%) (**Table 3**). Among the other 33 immunocompromised cases, organ transplantation was the most common, with twelve (32.4%) cases of solid organs and stem cell transplantation, followed by six (16.2%) cases of leukemia, and three (8.1%) cases of chronic ambulatory peritoneal dialysis (**Table 2**). Intestinal neutropenia does not appear to be an important risk factor for protothecosis. In our review, only 5 of the 14 patients for whom neutrophil counts were available showed neutropenia. Additionally, only two cases of AIDS were found, which is consistent with previous studies and suggests that a type of immunodeficiency other than AIDS contributes to susceptibility to protothecosis (12, 19). Interestingly, the case of CARD9 deficiency caused by *P. zopfii* provides a new insight into the mechanism of anti-Prototheca immunity (34). All cases of AIDS and diabetes are related to *P. wickerhamii*, whereas transplantation and leukemia do not seem to be associated with the species type. However, more data are needed to prove the relevance of this association (**Table 3**).

**Table 3:** Underlying conditions according to the species in 37 cases with disseminated protothecosis.

Underlying conditions	*P. wickerhamii*	*P. zopfii*	*Protoheca* spp.	Total
	n mortality (%)	n mortality (%)	n mortality (%)	n mortality (%)
Transplantation^†^	7 6(85.7)	4 4(100.0)	1 0(0.0)	12 10(83.3)
AIDS^††^	2 2(100.0)	0 0(0.0)	0 0(0.0)	2 2(100.0)
Leukemia	3 0(0.0)	3 3(100.0)	0 0(0.0)	6 3(50.0)
Diabetes mellitus	2 0(0.0)	0 0(0.0)	0 0(0.0)	2 0(0.0)
Skin trauma	1 0(0.0)	0 0(0.0)	0 0(0.0)	1 0(0.0)
Inherited CARD9 deficiency	0 0(0.0)	1 0(0.0)	0 0(0.0)	1 0(0.0)
Others^†††^	7 1(14.3)	0 0(0.0)	2 1(50.0)	9 2(22.2)
Healthy	3 0(0.0)	0 0(0.0)	1 0(0.0)	4 0(0.0)
Total	25 9(36.0)	8 7(87.5)	4 1(25.0)	37 17(45.9)

^†^Renal transplant, Lung transplant, Liver transplant, Cardiac transplant, and Stem cell transplant.

^††^One patient suffered AIDS and skin trauma.

^†††^Myasthenia gravis, Candidiasis, Anemia, Adenocarcinoma, CAPD.

In addition to one traumatic implant, 22 (59.5%) patients had a history of surgical and instrument injury related to defects in the skin and mucosa. Although glucocorticoid therapy might be considered the highest risk factor for *Prototheca* infection, it was not found among the patients reviewed (2). We believe this situation still exists because of the high proportion of organ transplantation cases and the one case of myasthenia gravis (15), in which large doses of glucocorticoids must be used for long periods. A likely explanation for the glucocorticoids as predisposing factors to infection may include exogenous or endogenous aspects. It may be on one hand to shrink and thin the epidermis to weaken the barrier function of the skin and, on the other hand, may suppress lymphocyte activation and impair PMNs and macrophages to increase endogenous colonization (46).

**Figure 1:**
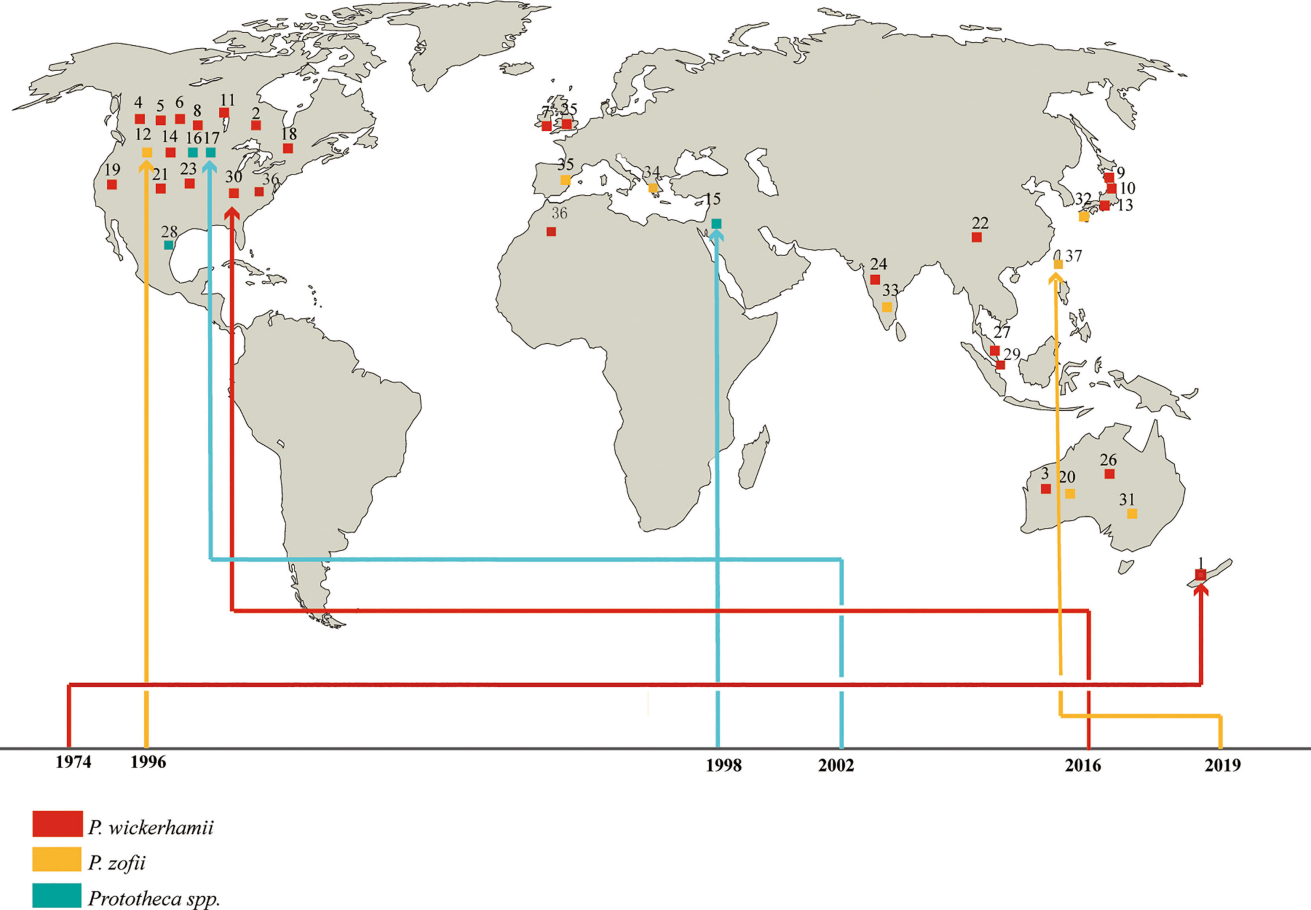
Geographic distribution of patients with disseminated protothecosis collected during the study period.

Protothecosis occurs globally and has been reported on every continent except Antarctica (2). In this review, the world distribution of 37 disseminated cases is shown in **Figure 1**. It predominated in the USA (fifteen, 40.5%), where it is prevalent in the southeast, followed by Australia and Japan (four each, 10.8%). Two of the cases were from England (5.4%), two from India (5.4%), two from China (5.4%), and one each from Israel, Malaysia, Singapore, Spain, Mexico, Turkey, Morocco, and New Zealand (2.7%). In general, *Prototheca* species seem to have a preference for warm and humid climates, which matches the epidemiology of the disease in both humans and animals. Similar to what has been reported in previous studies, we found that males are more affected (26 cases, 70.3%) compared to females (11 cases, 29.7%). Disseminated protothecosis can occur in individuals of any age, but mostly in patients between 30 and 60 years old. Median = 39 years (**Table 2**).

## Clinical Features

**Figure 2:**
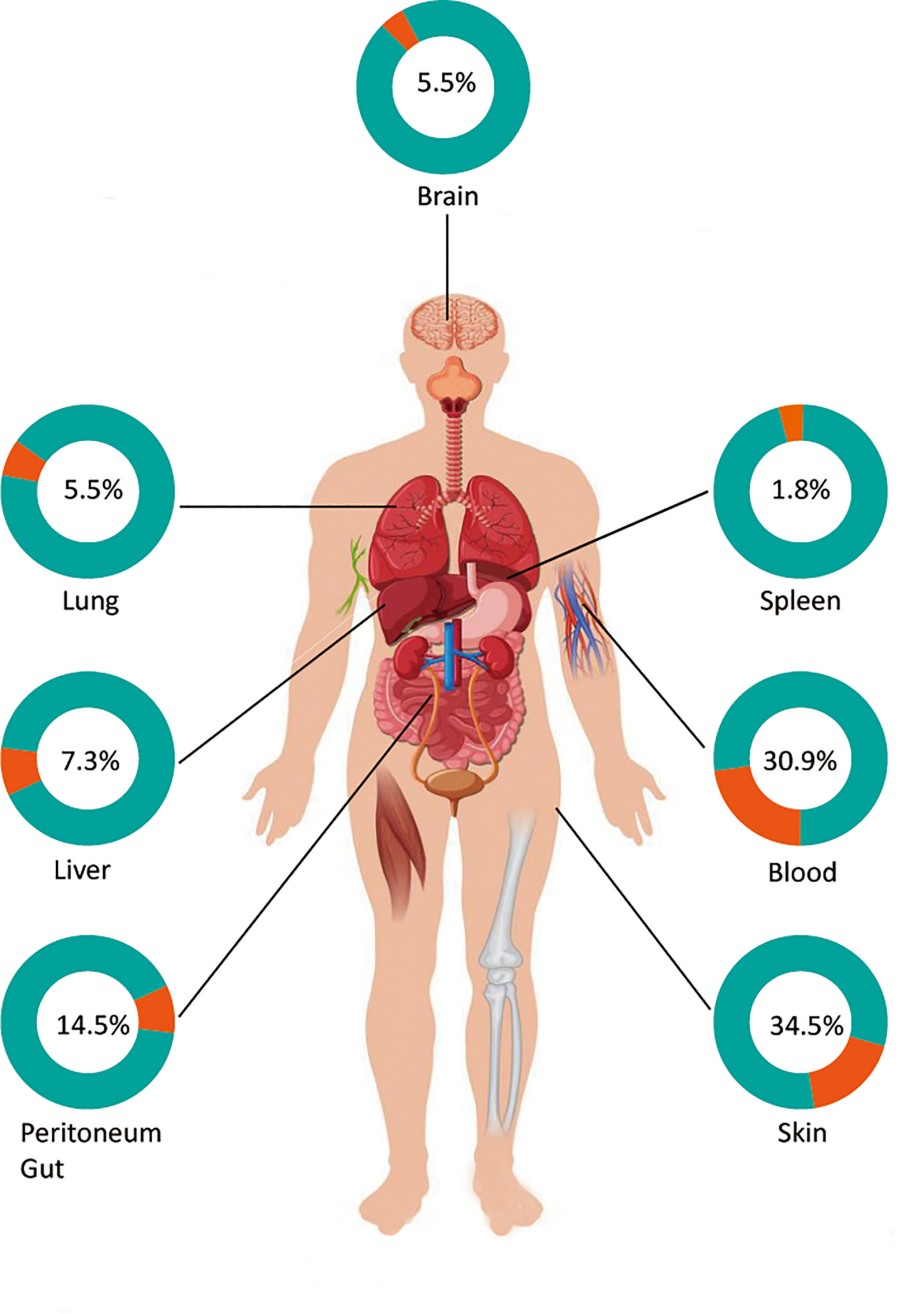
Main infectious sites of patients with disseminated protothecosis.

No specific clinical features were noted. Clinical initial signs of disseminated protothecosis in humans can include fever, skin lesions, abdominal pain, diarrhea, and headache, which are associated with the infectious sites. Similar to what has been reported in previous studies (2), the organs most commonly affected in dissemination are the skin (19 cases, 51.4%) and blood (17 cases, 45.9%), followed by the gut (5 cases, 13.5%) and liver (4 cases, 10.8%), then the lungs (3 cases, 8.1%), peritoneum (3 cases, 8.1%), and brain (3 cases, 8.1%) (**Figure 2**). There are various forms of skin lesions without specificity that can be manifested as erythematous papules, plaques, nodules, ulcers, papules, necrotic crusts, pustules, and bullae with purulent discharge, even presenting as an eczematoid eruption (4, 15, 19, 25, 31). At least three patients had lesions at the site of catheter implantation in this review (23, 25, 37). Unfortunately, even though skin lesions are the most common initial signs of this disseminated type, the mortality of patients with visual lesions was not lower than that of other factors, suggesting that atypical lesions were indeed overlooked or misdiagnosed by clinicians. In addition, algaemia, which represents blood involvement, is more easily covered up and ignored by bacteremia and fungaemia (45), especially under the condition of administration of prophylactic systemic antifungals, patient discharge, death, or transfer to another clinical unit. In addition, cholestatic jaundice and hepatitis are also considered the typical clinical presentations of systemic protothecosis (2). The algae can even invade the brain, but interestingly, out of a total of three patients with brain infection, only one patient with AIDS died, and the remaining two patients had a good prognosis. One immunocompetent patient has been asymptomatic but alga has tested positive for cerebrospinal fluid for 7 years (14). In all but those three cases, the species involved was *P. wickerhamii*.

Disseminated protothecosis is rare but has a high mortality (17/37, 45.9%). The majority of patients are over 30 years of age or elderly (higher mortality, fourteen, 60.9%). Cases with organ transplants (10/12, 83.3%) have higher mortality compared to leukemia (3/6, 50.0%) and other diseases (4/19, 21.1%) (*P* <.05). Among all, *P. zopfii* patients showed a higher rate of mortality (7/8, 87.5%) than patients infected with *P. wickerhamii* (9/25, 36.0%), leading to 100% of deaths among transplant and leukemia patients (**Table 3**). We hypothesized that the cause of the high mortality in those groups might be associated with the central venous catheter or Hickman catheter, thereby increasing the opportunity for biofilm formation and contributing to hard-to-treat characteristics. In many cases of protothecosis, co-occurrence with other pathogens was found, such as *Candida*, *Staphylococci*, herpes simplex virus, *Enterococci*, *Leuconostoc*, *Klebsiella*, *Cryptococcus*, *Blastomyces dermatitidis*, *Pseudomonas*, or *Escherichia* (4, 8, 10, 12, 16, 23, 25, 27, 33, 36). Our data showed that there were eleven (6/11, 54.5%) co-infected patients with slightly higher mortality than those (11/26, 42.3%) who were not co-infected.

## Diagnostic and Therapeutic Challenge

**Figure 3:**
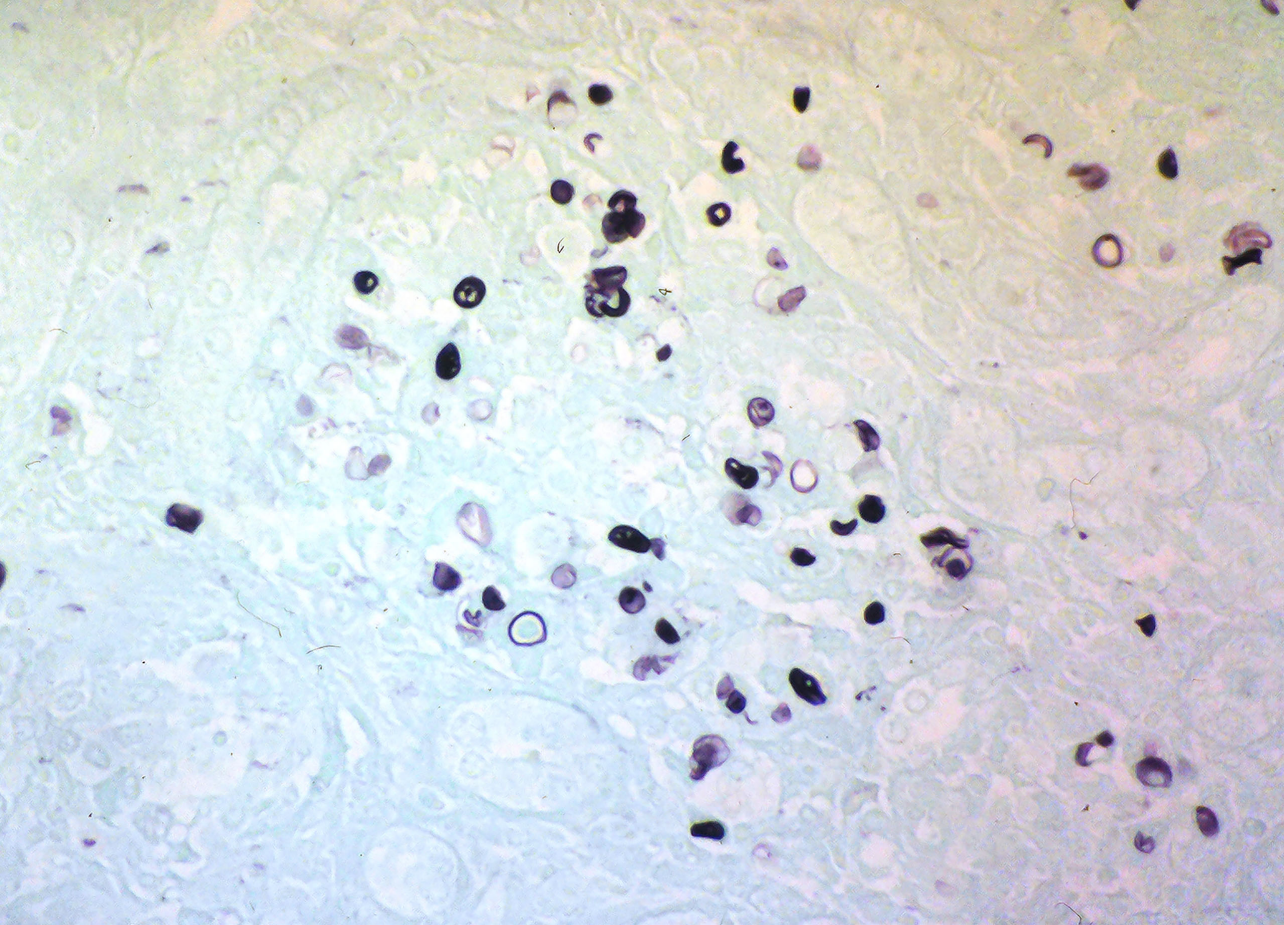
Grocott’s methenamine silver stain of histology section showing Prototheca sporangia and spores (courtesy of Prof. Henrik Jensen).

Prototheca remains a diagnostic and therapeutic enigma. For the diagnosis, combining histopathological and microbiological tests is recommended for cases where protothecosis is suspected. Histopathologic examination of infected tissue may be accomplished using the PAS, GMS, or Gridley fungus stain to visualize the endosporulating sporangia (morula form) of *Prototheca* spp. (**Figure 3**). In addition to the size differences noted previously, the two species of *Prototheca* differ in that *P. wickerhamii* tends to form symmetrical morula forms, whereas these forms are rare in *P. zopfii*, which exhibits more random internal segmentation (2). However, in the absence of these morphological features, the organism may resemble other fungi such as *Blastomyces*, *Coccidioides*, *Cryptococcus*, *Emergomyces*, *Paracoccidioides*, *Pneumocystis*, *Rhinosporidium*, and chromoblastomycosis agents (2). Of note, diseases caused by these fungi differ clinically from protothecosis in the presence of respiratory symptoms since the infection is mainly acquired by inhalation. However, chromoblastomycosis is an implantation mycosis but has a chronic nature and is characterized by the presence of darkly pigmented muriform cells in the infected tissue.

**Figure 4:**
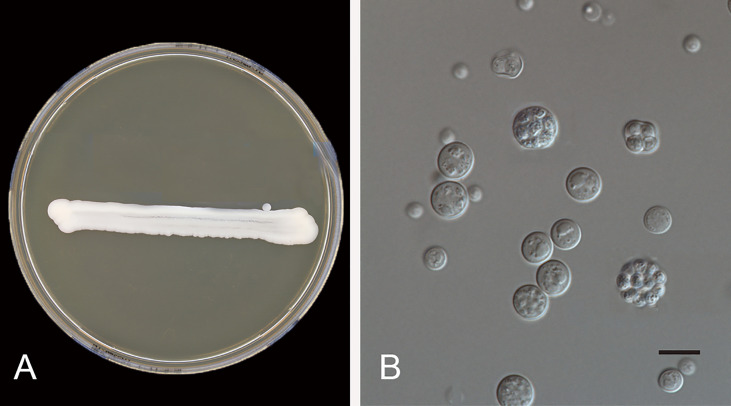
*Prototheca wickerhamii* (CBS 608.66). **(A)** Colonies on MEA 5 days at 24°C; **(B)** yeast-like organisms, the mature sporangium contains 2–20 or more autospores, and ruptures to release the daughter cells. Scale bars: 10 μm.

The inflammatory response in protothecosis is predominantly granulomatous but can consist of lymphocytes, plasma cells, eosinophils, neutrophils, macrophages, epithelioid cells, and giant cells. *Prototheca* species grow rapidly on routine laboratory media such as Sabouraud’s glucose agar, blood culture bottles, and blood agar (47). For confirmation, the presence of unicellular organisms, 3–30 µm in diameter, and sporangium containing autospores is indicative of *Prototheca* infection (**Figure 4**). Nevertheless, situations such as contamination of growth by co-infecting yeasts or bacteria, or growth of a single colony on solid media, often lead to missed diagnosis (45).

**Figure 5:**
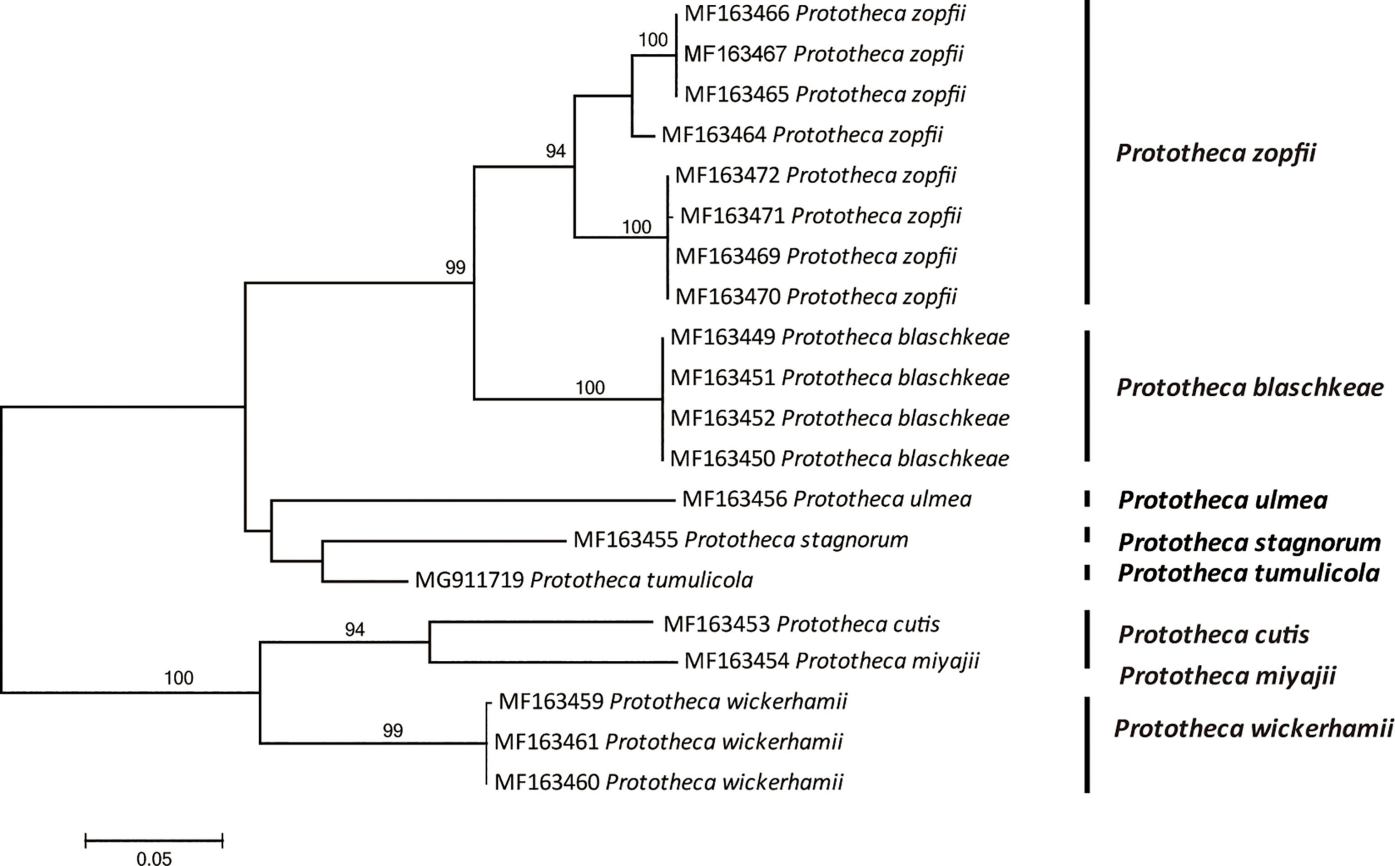
Phylogenetic tree constructed through maximum likelihood analysis based on *cytb* sequences. The bootstrap values obtained by the analysis are marked at the nodes.

There are possible differences in pathogenicity and treatment between *P. wickerhamii* and *P. zopfii*, so the identification of these algae to species level has become an inevitable trend. In addition to the traditional morphological methods, commercial physiological systems such as API 20C or API 20C-AUX and the database of VITEK 2 have been developed (48). Currently, rapid automated identification of *Prototheca* is possible using matrix-assisted laser desorption ionization–time-of-flight mass spectrometry (MALDI-TOF MS) (49). In addition, the sequencing of 18S and 28S rDNA has been applied for the identification and genotyping of species (50, 51). Recently, the mitochondrial *cytb* gene has been proved effective for discrimination and suggested as the gold standard for the identification of the *Prototheca* microalgae (40) (**Figure 5**).

Therapeutically, in addition to the fact that algae in general have low susceptibility to antimicrobial agents, there has been no consistency in the clinical responses. Treatment of protothecal infections remains a challenge (32). Antifungals such as amphotericin B and itraconazole form the mainstay of treatment, although *Prototheca* is susceptible to voriconazole, miconazole, clotrimazole, tetracycline, gentamicin, amikacin, and polymyxin B (29). Disseminated patients treated with an antifungal regimen that included amphotericin B were more likely to survive than those treated with a triazole alone. Amphotericin B and its lipid-based formulations provide broad spectrum cover, however, treatment failures even with combination antifungal therapy with amphotericin B have been reported (46). Our data also suggest that amphotericin B is effective in only 56.5% (13/23) of the disseminated patients. Antifungal treatment needs to be reassessed in cases of no clinical improvement. Furthermore, catheter removal should be the first consideration in treating a catheter-related *Prototheca* infection.

## Conclusions and Prospect

Human Disseminated Protothecosis is an emerging environmental algal disease with high mortality that typically occurs in immunocompromised individuals. Under the condition of immune deficiency, the destruction of the skin barrier caused by surgery and catheter is highly considered to be associated with this type. Organ transplantation is the most common risk factor, followed by leukemia. *P. wickerhamii* and *P. zopfii* are the dominant species that cause disseminated infection. The former has a significantly lower mortality than the latter, but is associated with brain infections. Low susceptibility to antimicrobial agents and *Prototheca* biofilms contributes to the hard-to-treat character of this algal infection.

Significantly, our study confirmed that disseminated protothecosis most frequently involves the skin, which is indeed different from other opportunistic biofilm-forming fungi prevalent in intensive care units (ICUs) and transplant patients. *Candida auris*, for example, is usually associated with bloodstream infections rather than skin infections (52). The difference in clinical manifestations could be related to the biological behavior of the species. The dominant species *P. wickerhamii*, which causes disseminated protothecosis, has an optimum growth temperature between 25 and 37°C and cannot grow above 40°C (53), however, *C. auris* could grow well at 42°C (52). Based on the difference in this thermal tolerance property, it is hypothesized that the skin is a window through which *Prototheca* spp. diffuses back into the environment to escape the powerful thermoregulatory immunity of the body. Since this study has limitations due to the small sample, more research work, especially the association of each species type with distinct profiles of clinical manifestation and response to treatment and epidemiological patterns, should be launched.

## Data Availability Statement

The original contributions presented in the study are included in the article. Further inquiries can be directed to the corresponding authors.

## Author Contributions

XW and YR carried out the literature search and participated in the data analysis and drafted the manuscript. YaJ designed this project, phylogenetic tree construction, participated in the data analysis, and revised the manuscript. SJ and XL carried out the statistical analysis. SA and YiJ contributed to the discussion and revision of the manuscript. All authors listed have made a substantial, direct, and intellectual contribution to the work and approved it for publication.

## Funding

This work was supported by the District Science Foundation Program (NSFC No. 81960368) from the National Natural Science Foundation of China, the Guizhou Provincial Natural Science Foundation [ZK (2021) zhongdian030], and the Doctoral Cultivating Fund of Guizhou Medical University (gyfybsky-2021-56).

## Conflict of Interest

The authors declare that the research was conducted in the absence of any commercial or financial relationships that could be construed as a potential conflict of interest.

## Publisher’s Note

All claims expressed in this article are solely those of the authors and do not necessarily represent those of their affiliated organizations, or those of the publisher, the editors and the reviewers. Any product that may be evaluated in this article, or claim that may be made by its manufacturer, is not guaranteed or endorsed by the publisher.

## Abbreviations

CARD9, caspase recruitment domain-containing protein 9; AIDS, Acquired Immune Deficiency Syndrome; PMN, polymorphonuclear; PAS, periodic acid Schiff reaction; GMS, Gomori methenamine silver stain; MALDI-TOF MS, Matrix-Assisted Laser Desorption/Ionization Time of Flight Mass Spectrometry.
